# Supplementary material for: Venomix: a simple bioinformatic pipeline for identifying and characterizing toxin gene candidates from transcriptomic data
Source: PeerJ. 2018 Jul 31;6:e5361. doi: 10.7717/peerj.5361 (PMC6074769; doi:10.7717/peerj.5361)
Supplement: Supplemental Information 3 [file peerj-06-5361-s003.gz › FinalOutput_E-6/Astacin-like_metalloprotease_toxin_3_2/finaltree.pdf]

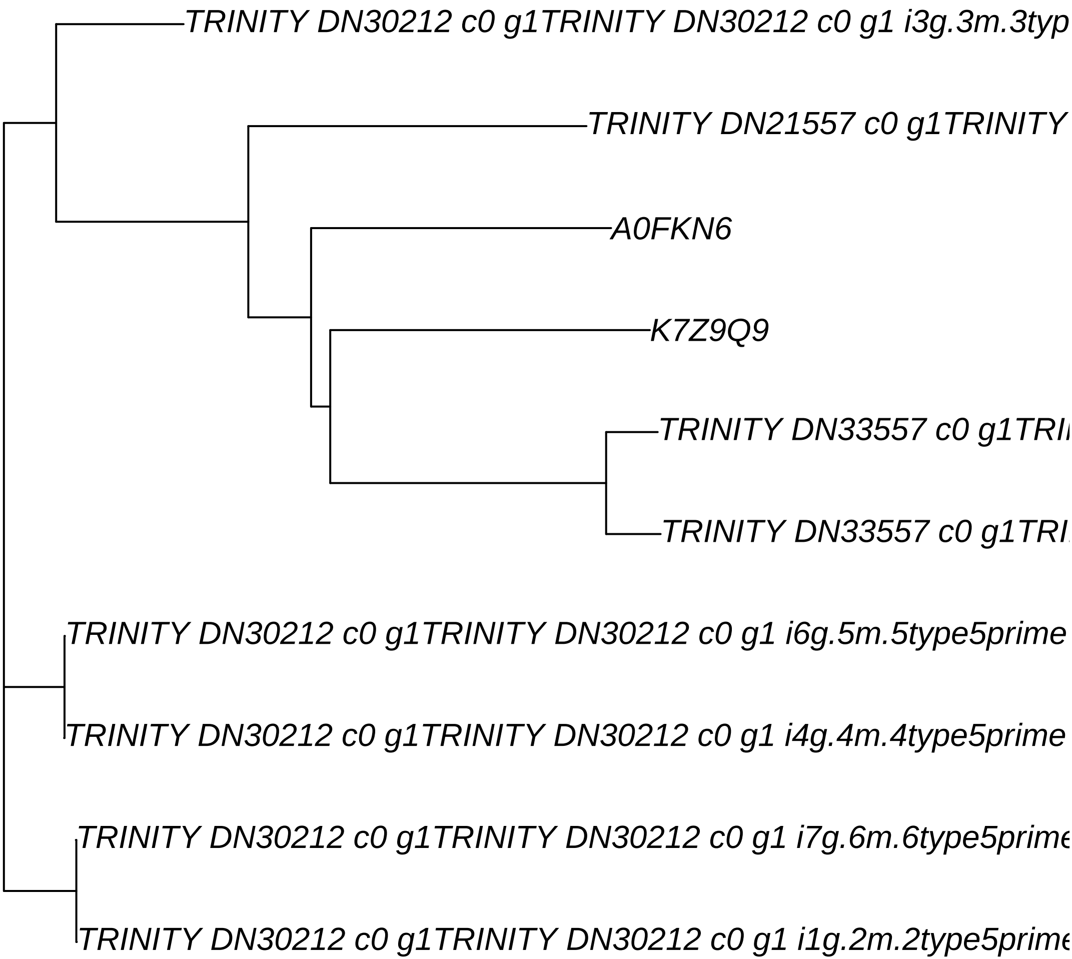
TRINITY DN30212 c0 g1TRINITY DN30212 c0 g1 i3g.3m.3typ
TRINITY DN21557 c0 g1TRINITY
A0FKN6
K7Z9Q9
TRINITY DN33557 c0 g1TRI
TRINITY DN33557 c0 g1TRI
TRINITY DN30212 c0 g1TRINITY DN30212 c0 g1 i6g.5m.5type5prime
TRINITY DN30212 c0 g1TRINITY DN30212 c0 g1 i4g.4m.4type5prime
TRINITY DN30212 c0 g1TRINITY DN30212 c0 g1 i7g.6m.6type5prime
TRINITY DN30212 c0 g1TRINITY DN30212 c0 g1 i1g.2m.2type5prime
